# Stromal Lineage Precursors from Rodent Femur and Tibia Bone Marrows after Hindlimb Unloading: Functional Ex Vivo Analysis

**DOI:** 10.3390/ijms24108594

**Published:** 2023-05-11

**Authors:** Elena Markina, Elena Andreeva, Ludmila Buravkova

**Affiliations:** Institute of Biomedical Problems, Russian Academy of Sciences, 123007 Moscow, Russia; goncharova-tim@list.ru (E.M.); buravkova@imbp.ru (L.B.)

**Keywords:** microgravity simulation, hindlimb unloading, tibia, femur, bone marrow, multipotent mesenchymal stromal cells, CFU-f, osteodifferentiation, transcriptomics

## Abstract

Rodent hindlimb unloading (HU) model was developed to elucidate responses/mechanisms of adverse consequences of space weightlessness. Multipotent mesenchymal stromal cells (MMSCs) were isolated from rat femur and tibia bone marrows and examined ex vivo after 2 weeks of HU and subsequent 2 weeks of restoration of load (HU + RL). In both bones, decrease of fibroblast colony forming units (CFU-f) after HU with restoration after HU + RL detected. In CFU-f and MMSCs, levels of spontaneous/induced osteocommitment were similar. MMSCs from tibia initially had greater spontaneous mineralization of extracellular matrix but were less sensitive to osteoinduction. There was no recovery of initial levels of mineralization in MMSCs from both bones during HU + RL. After HU, most bone-related genes were downregulated in tibia or femur MMSCs. After HU + RL, the initial level of transcription was restored in femur, while downregulation persisted in tibia MMSCs. Therefore, HU provoked a decrease of osteogenic activity of BM stromal precursors at transcriptomic and functional levels. Despite unidirectionality of changes, the negative effects of HU were more pronounced in stromal precursors from distal limb—tibia. These observations appear to be on demand for elucidation of mechanisms of skeletal disorders in astronauts in prospect of long-term space missions.

## 1. Introduction

Long-term spaceflights adversely affect the musculoskeletal system, primarily the bones [1,2,3]. Aboard rodent experiments revealed a reduction of bone tissue mineralization, a decrease of alkaline phosphatase and an increase of acid phosphatase activity, an inhibition of periosteal remodeling in tubular bones [4]. 

The probable cause may involve the damage of stromal lineage cells which provide physiological and reparative remodeling of skeletal tissues. Cells of mesenchymal stromal lineage play an important role in providing homeostasis and physiological remodeling of various tissues, primarily the musculoskeletal system [5].

According to current views, mesenchymal stem cells (MSCs) undergo proliferative and commitment events, and their progeny enter discrete lineages that result in the ultimate terminal differentiation of definitive phenotypes such as osteoblasts, chondroblasts, or myoblasts [6]. Thus, the stromal compartment consists of cells of different commitment, among which the population of primitive precursors localized in the bone marrow (BM) is of particular interest. Multipotent mesenchymal stromal cells (MMSCs) can be isolated from bone marrow (BM). Ex vivo, MMSCs represent a heterogeneous population consisting of stromal precursors of different commitment including fibroblast colony-forming units (CFU-f) mostly close in their functionality to mesenchymal stem cells (MSCs) [7].

To date, a set of ex vivo methodological approaches has been established, allowing to evaluate the potential of CFU-f and MMSCs [8].

After spaceflights, a decrease in the number of CFU-f in the rat BM was noted after Bion biosatellite flights [9]. At the same time, no negative effects on CFU-f number or proliferative activity of MMSCs from mice BM were detected after Bion-M1 experiment [10]. A decrease in the number of osteoblasts in rat bones was described in Cosmos biosatellite missions [11,12]. Significant downregulation of genes encoded osteoproteins were in detected stromal precursors from rat femur in SLS-2 experiment [13]. 

With the prospect of interplanetary missions, the distance and duration of space flights will only increase, so the data from long-term ground-based simulations of spaceflight effects are in high demand. In the 1980s, the rodent hindlimb unloading (HU) model was developed to make it possible to study mechanisms, responses, and treatments for the adverse consequences of microgravity. Currently, HU is considered as one of the most appropriate analogs of spaceflight factors as a load deprivation and a headward shift of internal organs and fluid [14]. The effects similar to spaceflight were found after rodent HU: decreased bone mass of hindlimbs, negative calcium balance and decreased bone density [15]. After HU, the decrease in the number of osteoblasts was found, as was downregulation of genes encoding bone matrix proteins involved in the maintenance of bone tissue homeostasis [16]. In mice HU, during a period of 4 weeks, the effects on bone and muscle gene expression was followed, demonstrating the time-dependent regulation of genes encoded structural and regulatory proteins. Irisin treatment prevented negative consequences of unloading [17]. In vitro simulation using Random Positioning Machine (RPM) needs to be mentioned in respect of stromal lineage fate under microgravity. As it was demonstrated, RPM exposure provoked morphological and metabolic alterations of human osteoblasts and murine MC3T3-E1 cells characterized by increased levels of reactive oxygen species, slowdown of the proliferative rate, or even cell death, mineralizing capacity loss, while Irisin or antioxidant Trolox could prevent this damage [18,19]. Above mentioned data indicate the potency of Irisin and antioxidants to counteract the musculoskeletal impairment caused by unloading.

It is reasonable to assume that the changes after microgravity simulations like RPM and HU can be provoked by alterations in BM stromal lineage cells including primitive and committed precursors. The number and activity of stromal precursors in BM can differ in proximal and distal parts of the hindlimb skeleton, which will determine susceptibility to the negative effects of microgravity. 

In this study, we evaluated ex vivo the influence of space environment simulation in HU model on the stromal lineage precursors from the femur and tibia bone marrows after 14 days of HU and subsequent 14 days of reloading (RL).

## 2. Results

### 2.1. Bone Marrow (BM) Cellularity 

The number of total nucleated cells (TNC) was calculated after isolation of BM from the femur and tibia bones of the control and experimental animals. In the control group, the number of TNCs from the femur bones was, on average, 1.5 times higher than in the tibia bones. After HU, the cellularity significantly decreased in both bones: 2.2 and 1.7-fold in the femur and tibia, respectively. There was no complete recovery of TNC number during the 14 days of reloading (RL); there were still significantly fewer cells in both bones than in the VC. Only in the tibia, the number of TNCs after RL significantly increased compared to HU (Table 1). 

### 2.2. The Ex Vivo Analysis of Stromal Progenitors from Femur and Tibia BM

It is well known that the population of stromal progenitors in BM is heterogeneous. It includes multipotent mesenchymal stromal cells (MMSCs) as well as their primitive precursors (CFU-f) and more committed progeny. The potentials of these cells can be evaluated ex vivo using well established methodological approaches.

#### 2.2.1. Fibroblast Colony-Forming Units (CFU-f) 

**Figure 1:**
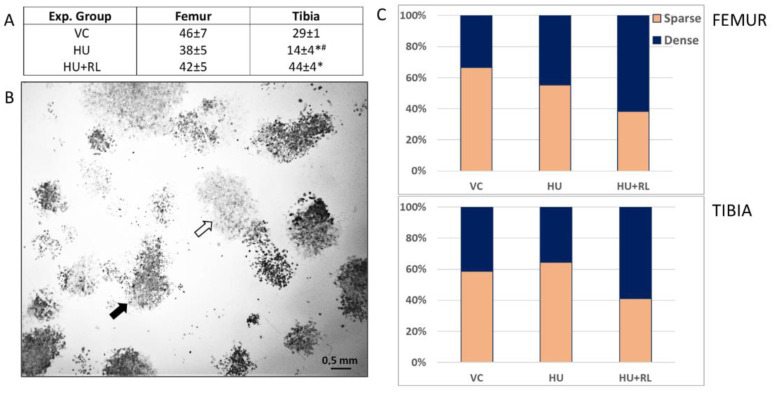
Characterization of CFU-f from rat hindlimb bone marrow. CFU-f number among rat hindlimb bone marrow TNCs. The data are presented as mean ± SEM of CFU-f per 4 × 10^5^ TNCs (*n* = 3). * *p* < 0.05, the significant difference from VC, ^#^ *p* < 0.05, the significant difference from HU + RL. (**A**). CFU-f-derived colonies after ALP activity identification and Neutral Red counterstaining, representative image. Black arrow indicates a dense colony, white arrow points a sparse colony, (**B**). The proportion of sparse/dense colonies in rat hindlimb bone marrows, (**C**). VC—Vivarium Control; HU—Hindlimb Unloading; HU + RL—Reloading after HU.

The clonal growth capacity at low plating density is one of the most reliable traits to identify primitive stromal precursors known as colony-forming fibroblast units (CFU-f) [20,21]. BM TNCs were seeded in primary culture at limiting density of 4 × 10^4^ cells/mm^2^, as described previously [22]. After 14 days, colonies of fibroblast-like cells could be seen. Each of these colonies was formed as a result of the proliferation of a single CFU-f (Figure 1).

In the VC group, the frequency of CFU-f in femur exceeded tibia values almost twice. HU and subsequent RL did not affect the number of CFU-f in the femur. In the tibia, the frequency of CFU-f after HU decreased twofold. After RL, the number of colonies in the tibia was 3 times higher than after HU and 1.5 times higher than in the VC group (Figure 1A).

The CFU-f colonies were heterogeneous in morphology and size: some of them were loose, weakly stained, with well distinguished single cells, while others were dense, intensely stained, the single cells were undistinguishable (Figure 1). As it was demonstrated earlier, the colonies’ appearance reflects the ability of CFU-f to proliferate. Actively proliferating clones were characterized by compact packaging of cells (dense colonies), while slow growing clones look loose with isolated cells [23].

RL after HU resulted in an increase of the dense colonies ratio among femur-derived CFU-f comprising about 2/3 of total colonies. Taking into account that dense colonies are composed by actively proliferating cells, one can conclude that unloading stimulated proliferation of femur BM CFU-f and that this process continued after reloading.

Multilineage differentiation, including osteogenic, is an intrinsic feature of CFU-f progenitors. We examined the spontaneous osteogenic activity in the CFU-f colonies. For this purpose, the activity of alkaline phosphatase (ALP), a marker enzyme of early osteocommitment, was assessed histochemically. The area of stained cells containing the product of the enzyme activity (ALP+) and those without (ALP−) was determined. Both in the VC and after HU/RL, the squares of ALP+ cells were about 60% in CFU-f derived colonies from the BM of both bones.

Thus, CFU-f from the tibia were sensitive to support deprivation, which manifested as a decrease of their frequency. Restoration of the support resulted in an even higher number of CFU-f than in the VC group. In addition, the stromal progeny of CFU-f displayed higher proliferative activity (more dense colonies) than the cells from the femur. These data suggest a higher plasticity of CFUs-f from the tibia.

#### 2.2.2. Multipotent Mesenchymal Stromal Cells (MMSCs)

As it was mentioned above, retrospective assessment of the functional activity of MMSCs in vitro is currently the only approach to study the mechanisms of the involvement of these cells in physiological and reparative remodeling in vivo [24]. Due to the fact that the number of MSCs in the body is very limited, expansion of these cells is necessary to obtain a sufficient amount for examination. To obtain MMSCs, BM TNCs from the femur and tibia BM were seeded in standard density and cultured until the fibroblastlike cells reached the PR monolayer (70%–80% of confluency). Cells were passaged once to escape from the admixture of hematopoietic cells. The remaining MMSCs were subculture with the desired density according to the protocols of the experiments. The functional state of the MMSCs was analyzed after the first passage.

##### Cell Growth

MMSC proliferative activity was assessed as the number of population doublings (PD) during time period from day 4 to day 14 (Table 2). The PD of MMSCs from femur and tibia were approximately the same in VC and decreased significantly after HU. The proliferative potential of the MMSCs was completely restored after 2 weeks of RL, indicating the activation of the MMSC proliferation.

##### MSC Osteogenic Potential

To characterize the osteogenic potential of MMSCs the initial level of osteocommitment and the osteogenic capacity in response to appropriate inductive stimuli were examined. 

**Figure 2:**
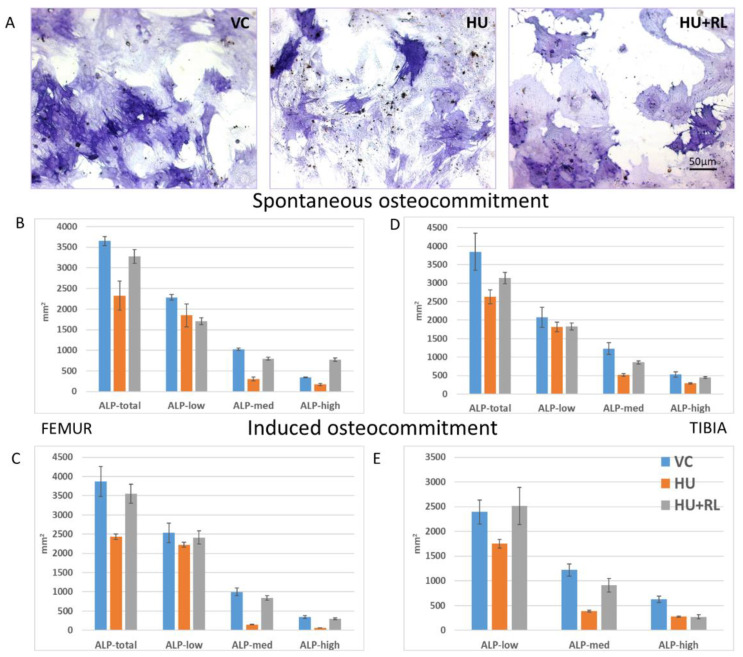
Osteogenic activity of MMSCs from rat hindlimb bone marrows. ALP activity evaluation, representative images, (**A**). Semiquantitative analysis of ALP activity in MMSCs. The data are presented as squares occupied with MMSCs varied in ALP activity: cells with low ALP activity—ALP-low; cells with medium ALP activity—ALP-med; cells with high ALP activity—ALP-high. Spontaneous osteo commitment without osteoinduction (**B**,**D**); induced osteo differentiation in the presence of osteogenic stimuli (**C**,**E**). VC—Vivarium Control; HU—Hindlimb Unloading; HU + RL—Reloading after HU.

Early stages of spontaneous osteocommitment or induced osteodifferentiation were assessed using histochemical evaluation of ALP activity. Subsequently, the staining intensity of the reaction product was evaluated semiquantitatively using Sigma Scan Pro 5 software. For this purpose, the area occupied by stained cells were measured on microphotographs of randomly selected view fields as shown in Figure 2.

The averaged staining intensity of ALP reaction product in the cultured MMSCs was approximately the same in nonstimulated (spontaneous commitment) and osteoinduced MMSCs in VC groups and fell into channels from 1 to 80. After HU, the intensity of staining decreased almost twice. The enzyme activity completely recovered after two weeks of restoration of loading support. In tibia MMSCs, the response to osteoinduction in RL group was significantly more pronounced than in VC.

Morphological analysis showed that both nonstimulated and osteoinduced MMSC populations were heterogeneous in terms of ALP activity (Figure 2B–E). MMSCs were grouped according to the staining intensity: cells with low ALP activity—ALP-low (channels 1–20); cells with medium ALP activity—ALP-med (channels 21–40); cells with high ALP activity—ALP-high (channels 41–80). As shown in the histograms, most of the MMSCs in VC, HU, and RL groups were ALP-low. The area occupied by ALP-low MMSCs was about 50% or more of the total area of stained cells. Regardless of osteoinduction, decrease in the stained MMSC square after HU was due to the decrease in ALP-med MMSCs from both bones.

Thus, the MMSCs from the femur and tibia BM showed similar levels of ALP activity and demonstrated the same responses to deprivation of support load revealed as a decrease of ALP activity. The recovery of ALP activity after RL was more pronounced in MMSCs from the tibia.

**Figure 3:**
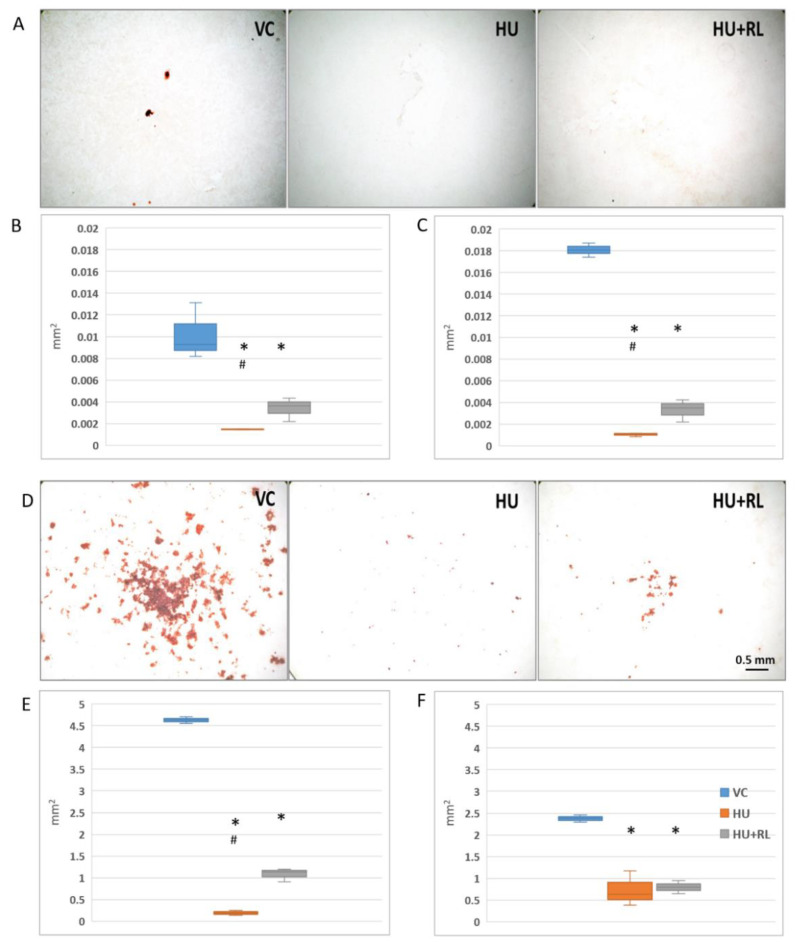
Mineralization of extracellular matrix in MMSC cultures from rat hindlimb bone marrows. Mineralized extracellular matrix stained with Alizarin Red, representative images of femur-derived MMSCs (**A**,**D**). Semiquantitative analysis of mineralized matrix deposition. The data are presented as mean + SEM of squares occupied with stained matrix, *n* = 3. Spontaneous osteo-commitment without osteo-induction (**B**,**C**); induced osteo-differentiation in the presence of osteogenic stimuli (**E**,**F**). * *p* < 0.05, the significant difference from VC. ^#^ *p* < 0.05, the significant difference from HU + RL. VC—Vivarium Control; HU—Hindlimb Unloading; HU + RL—Reloading after HU.

The mineralization of ECM, as a sign of late stages of MMSC osteodifferentiation, was examined semiquantitatively after Alizarin Red staining. The areas occupied by mineralized ECM were calculated with Sigma Scan Pro (Figure 3). 

ECM mineralization in unstimulated MMSCs was low (Figure 3A). In tibia MMSCs, the level was twice as high as it was in the femur. HU led to a dramatic decrease in mineralization efficacy in MMSCs from both BM. After RL, the area of mineralized ECM increased significantly but without reaching the values in VC (Figure 3B,C).

In comparison with unstimulated MMSCs, the areas of mineralized ECM after osteo-induction in the VC increased by 400 and 100 times in MMSCs from the femur and tibia, respectively (Figure 3D–F). After HU, as well as in unstimulated MMSCs, a dramatic suppression of ECM mineralization with incomplete recovery after support restoration was detected. 

Thus, HU significantly inhibited the late stages of MMSC osteo-differentiation associated with ECM mineralization, and it was manifested both in unstimulated and osteoinduced cells. Tibia MMSCs demonstrated more pronounced spontaneous ECM mineralization. Apparently, it might determine their weaker response to osteoinduction. In contrast to ALP activity, there was no restoration of the initial level of ECM mineralization during 14 days of RL. It can be assumed that it is the late stages of bone tissue osteoremodeling that may be susceptible to the negative effects of support unloading.

##### Transcriptomic Activity

**Figure 4:**
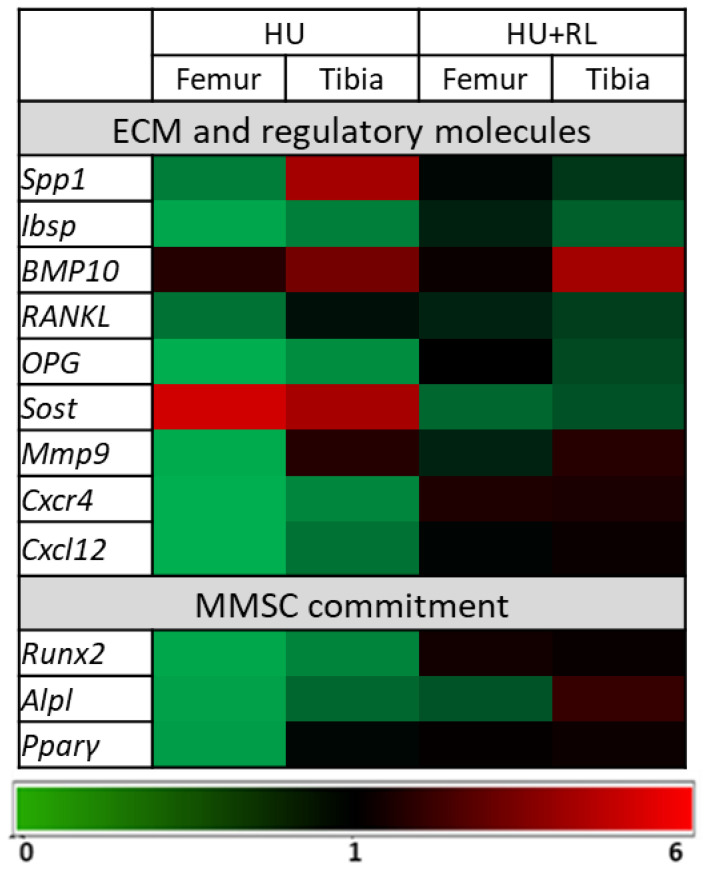
Differential expression of genes encoded extracellular matrix (ECM), regulatory and commitment-related molecules in MMSCs from hindlimb bone marrow. The data are presented as fold changes of transcription levels from VC, (*n* = 3). VC—Vivarium Control; HU—Hindlimb Unloading; HU + RL Reloading after HU.

The differential expression of genes encoding molecules involved in MMSC stromal functions (*Mmp9*, *Spp1*, *Cxcl12*, *RANKL*, *OPG*, *Ibsp*, *BMP10*, *Sost*), interaction with HSCs (*Cxcl12*), and MMSC commitment (*Pparγ*, *Runx2*, *Alpl*) was examined (Figure 4).

In both tibia and femur MMSCs, almost all genes responsible for bone tissue homeostasis: bone ECM genes (*RANKL*, *OPG*, *Ibsp*) osteo- (*Runx2*, *Alpl*) and adipodifferentiation (*Pparγ*), ECM remodeling (*Mmp9*) was downregulated after HU. The genes encoding Cxcl12/Cxcr4 axis of MMSC/HSC interaction were downregulated as well. *Sost* (sclerostin) transcript level was increased in the MMSCs from both bones. *Spp1* (osteonectin) and *BMP10* were upregulated in MMSCs from the tibia only.

After RL, the activity of most examined genes in femur MMSCs was similar to VC. The only exception was detected for downregulated *Sost* and *Alpl*. In MMSCs from both bones, a decrease of *SOST* (sclerostin) transcript levels was detected. In MMSCs form tibia BM, the altered transcription of bone matrix genes (*OPG*, *Ibsp*, *Spp1*, *BMP10*) was persisted. *Sost* was downregulated as in femur MMSCs as well. 

Thus, in MMSCs from femur and tibia BM, HU provoked the changes of transcription profiles of genes encoding bone matrix proteins and regulatory molecules. These genes were mainly downregulated, which could determine the above-described suppression of MMSC osteopotential. RL provided almost complete restoration of gene activity in the MMSCs from the femur but not the tibia BM.

##### Paracrine Activity

Soluble mediators with pro- or anti-inflammatory activity (IL-1a, IL-4, IL-1b, IL-2, IL-6, IL-13, IL-10, IL-12p70, IFNg, IL-5, IL-17A, IL-18, IP-10, TNFa) as well as bone metabolic regulators (ACTH, OPG, Insulin, Leptin, PTH, DKK1, SOST, FGF23) were analyzed in conditioned media of MMSCs from femur and tibia BM. Some of them were poorly represented—the mediators were either not detected or detected at the method sensitivity threshold. The most represented cytokines were as follows: IL-6, IL-13, IL10, IL-5, IP-10, TNF-a, and regulators of bone homeostasis: OPG, SOST (Table 3).

In VC groups, the levels of cytokines in conditioned media were close in femur and tibia MMSCs. The pattern of mediator changes after HU were similar as well. The levels of IL-6, IL-10, and TNF-a increased after HU and recovered to VC levels after RL. IP-10 concentration increased after HU and did not return after RL. Osteoprotegerin (OPG) and sclerostin (SOST) levels decreased after HU and restored after RL, exceeding VC values in the case of osteoprotegerin.

In whole, the change of the paracrine profiles after HU was characterized by elevation of proinflammatory cytokines (IL-6, TNF-a), accompanied by an imbalance in the signaling molecules, in particular, as evidenced by the increased production of IL-10. In addition, the expression of osteoprotegerin and sclerostin decreased, indicating that the bone tissue remodeling could be disturbed.

## 3. Discussion

Stromal BM precursors represent a source of replenishment of the osteogenic bone tissue cells [5]. The earlier data from our and others experiments demonstrate the unidirectional changes in bone tissue and BM stromal precursors after spaceflight and after ground-based simulation [4]. In the present paper, we intended to discriminate the differences between responses of MMSCs from BM of proximal and distal parts of hindlimbs to simulation of weightlessness.

A well-established rodent model for microgravity simulation—hindlimb unloading (HU)—was used in our research. HU was developed to explore musculoskeletal responses to the weightlessness and now is also used to investigate the muscle atrophy, disuse osteopenia, bed rest, and immobilization. The HU model provides the ability to examine response to load support restoration. The limitations of the HU are following from as inconsistencies in stress responses to HU depending on the experiment, continued gravitational loading of the forequarters, and lack of clarity about the influence of HU on the spine [14].

In HU, a decrease in hindlimb bone mass in mice and a decrease in bone mass and osteoblast number in rats [16] were described. The in vitro experiments revealed decreased proliferative activity of BM stromal cells, decreased number of CFU-f and inhibition of osteopotential of stromal precursors [25]. After HU, a downregulation of transcripts of osteocalcin (OS), bone sialoprotein (BSP), alkaline phosphatase (ALP) family proteins, bone morphogenetic protein 1 (BMP1), and stromal cell factor SDF-1/CXCL12 in stromal precursors from mouse BM were demonstrated [26]. 

After Cosmos/Bion biosatellite flights, it was supposed that the negative effects of microgravity are more pronounced in the distal parts of rodent hindlimbs [27]. To reveal possible molecular and cellular basis of these changes, we analyzed how two compartments of stromal precursors—CFU-f and MMSCs from the BM of rat femur and tibia bones were affected by deprivation and restoration of support loading. In control animals, the frequency of CFU-f among TNCs from tibia BM was two times lower than that from femoral BM. Only CFU-f from tibia BM were sensitive to HU/RL. Their number significantly decreased after HU and restored after RL, exceeding the VC values. Stromal progenitors of tibia CFU-f formed more dense colonies, which can indicate higher proliferative activity [23].

Previously, in HU experiments, Basso et al. detected a decrease in bone mass and number of osteoblasts after HU of rats [16]. The number of colony-forming units of fibroblasts (CFU-f) in BM was unchanged, while the alkaline phosphatase (ALP)-positive CFUs-f were diminished. In addition, the proliferative activity of stromal progenitors was decreased [16,28,29,30]. After short-term HU, the number of stromal progenitors with osteogenic potential decreased, whereas that of osteoclasts and adipocytes increased in the murine BM [31]. The prolonged HU led to the reduction of bone mass of murine hindlimbs [32]. Previously, we showed the negative effects of 30-day HU on the functional activity of BM progenitor cells of rodents. It was shown that HU of various durations (from 10 min up to 28 days) provoked alteration of the blood supply of rat BM as a result of vascular rearrangement, failure of endothelium dependent mechanism of vasodilation, and changes in bone structure. It was shown that HU of various durations (from 10 min up to 28 days) provoked alteration of the blood supply of rat BM as a result of vascular rearrangement, failure of endothelium dependent mechanism of vasodilation, and changes in bone structure [33,34,35]. This process worsened over time, the proliferative activity of stromal precursors was inhibited, and the number of CFU-f decreased [36]. In mice HU, the transcription of bone-related genes was downregulated, while Irisin treatment prevented negative consequences of unloading [17]. The above-mentioned data indicate the potency of HU for development of potential strategies for preventing disuse bone loss. 

We did not reveal any differences in the levels of spontaneous osteocommitment or response to osteoinduction among the CFU-f progeny and MMSCs from femur and tibia according to alkaline phosphatase activity considered as an early marker of osteocommitment. However, when analyzing the late sign of osteodifferentiation—the degree of extracellular matrix mineralization, we found that MMSCs from the tibia initially showed greater spontaneous mineralization of the ECM but were less sensitive to osteoinduction. In contrast to the alkaline phosphatase activity, there was no recovery of the initial level of ECM mineralization in the MMSCs from both BM during 14 days of RL. The negative influence of support deprivation on the late stages of bone remodeling can be evidenced.

In order to identify the possible regulatory factors underlying the detected differences, we analyzed the transcriptomic and paracrine activity of the cultured MMSCs. 

After HU, almost all genes related to bone tissue homeostasis (bone matrix proteins and their regulatory molecules) as well as osteo- and adipodifferentiation master genes were downregulated either in tibia or femur MMSC populations. Upregulation was detected only for the *Sost* in femur MMSCs and for *Sost*, *Spp1*, and *BMP10* in tibia MMSCs. After RL, the initial level of transcription was restored in the femur MMSCs, while suppression of expression of genes related to bone tissue homeostasis persisted in tibia MMSCs. 

The described changes in the transcriptional profiles may indicate disruption of the processes underlying bone tissue metabolism, in which the RANK/RANKL/OPG system and regulating inflammatory mediators (IL-1,-6,-11, TNF-a) play a significant role [37]. HU-mediated downregulation of *OPG*, *RANKL* and upregulation of *Sost* as well increased IL-6 and TNF-α levels in conditioned medium can be the case of the suppression of bone matrix gene expression.

To conclude, support unloading provoked a decrease of osteogenic activity of BM stromal precursors at the transcriptomic and functional levels. Despite unidirectionality of changes, the negative effects of HU were more pronounced in stromal precursors from distal limb—tibia, which may underline the greater sensitivity of these cells to unloading and the delayed response when support was restored. These observations appear to be on demand for understanding the mechanisms of development of skeletal disorders in astronauts in connection with the prospect of long-term space missions.

## 4. Materials and Methods

### 4.1. Animals and Procedures

**Figure 5:**
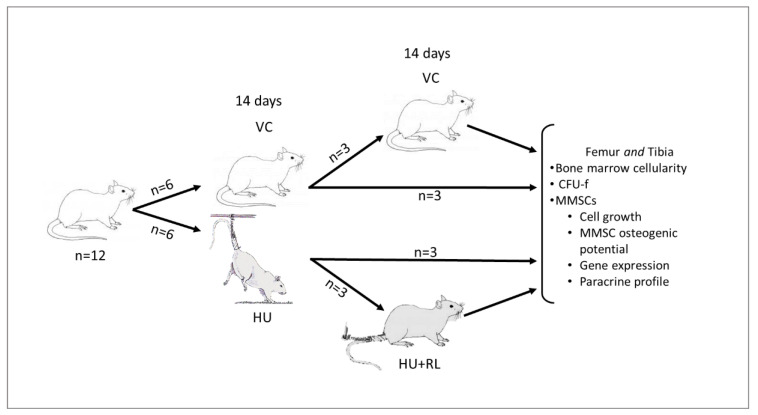
Study design. Male Wistar rats, 6 months of age, weighing 250–300 g, were divided into following groups: vivarium control (VC)—animals were kept in an animal house (*n* = 6), 14 days of hindlimb unloading (HU) (*n* = 3), reloading (RL)—14 days of load restoration after 14 days of HU (HU + RL) (*n* = 3). After the end of experiment, BM TNCs were isolated, enumerated, primary cultures were initiated for ex vivo assays.

The experimental design is presented in Figure 5. Twelve male Wistar rats, 6 months old, weighting 250–300 g, were randomly divided into following experimental groups: Vivarium Control (VC) (*n* = 6), Hindlimb Unloading (HU) (*n* = 6). During the experiment, the animals were kept in standard vivarium conditions and administered standard rodent food and water ad libitum (12-h day–night cycle). After 14 days, three animals in each group were selected randomly, sacrificed and BM specimens were prepared for further ex vivo analysis. The other three animals after HU were returned to the animal house. This group was marked as Hindlimb Unloading + Reloading (HU + RL). After 14 days, all animals (VC and HU + RL) were sacrificed and BM samples prepared for further examination. Rats were anesthetized via an intraperitoneal injection of 10% avertin solution at 5 mL/kg of body weight (Sigma-Aldrich Corp., St. Louis, MO, USA) and euthanized via cervical dislocation [38]. HU was executed according to well-established experimental protocol for small laboratory animals [39,40]. 

All procedures were approved by the Commission on Biomedical Ethics of the Institute of Biomedical Problems (IBMP) (Minutes No. 515 dated 10 June 2019).

### 4.2. Bone Marrow Cells Isolation and Enumeration 

Right femur and tibia bones were obtained from each animal. Bone marrow (BM) was isolated according to the generally accepted procedure in our modification [10]. Briefly, BM cells were flushed out from the tibia and femur bone cavities using a 10 mL syringe and 18-gauge needle filled with a-MEM with 2% FBS into 15 mL centrifuge tubes. After centrifugation at 500× *g* for 5 min, supernatants were removed cell pellets were resuspended in full culture medium. The viable nucleated cells were counted in a Neubauer chamber. The trypan blue exclusion test was applied to exclude dead cells.

### 4.3. Cell Cultures

All ex vivo assays were performed on freshly isolated cells. The cells of primary culture and first passage in dependence of the experimental design were used. The freezing/thawing procedures were not applied. 

BM cells were cultured in α-MEM supplemented with 2 mM glutamine (Gibco, Life Technology Ltd., Paisley, UK) containing 20% fetal bovine serum (HyClone laboratories, Logan, UT, USA), 100 units/mL penicillin, 100 µg/mL streptomycin (PanEco, Moscow, Russia), at 37 °C in an CO_2_ incubator (5% CO_2_, 100% humidity) (Sanyo Electric Co., Ltd., Moriguchi, Osaka prefecture, Japan). 

Cells were passaged when 80–90% of the confluence was reached in areas of high density of primary cultured cells. 0.25% trypsin-EDTA solution (Gibco, Life Technology Ltd., Paisley, UK) was used to detach the cells from the substrate. 

### 4.4. Fibroblast Colony-Forming Units (CFU-f)

To detect CFU-f, a suspension of BM cells was seeded at a limiting density of 4 *×* 10^4^ cells/cm^2^ in Ø 35 mm Petri dishes. On day 14, the CFU-f-derived colonies were fixed with 4% paraformaldehyde and alkaline phosphatase activity was detected histochemically using Alkaline Phosphatase Kit (Sigma-Aldrich Corp., St. Louis, MO, USA). Cells were counterstained with neutral red (Sigma-Aldrich Corp., St. Louis, MO, USA). Petri dishes with stained colonies were photographed using a Stemi 2000-C stereoscopic microscope (Carl Zeiss, Oberkochen, Germany). The colonies were counted. Further, the images were analyzed using Sigma Scan Pro 5 software (SPSS, Point Eichmond, CA, USA) to determine the total square of neutral red-stained cells and the square occupied with alkaline phosphatase-positive staining. The ratio of two parameters was used to characterize osteogenic potential of CFU-f progeny.

### 4.5. Proliferative Activity of MMSCs

To assess MMSC growth, BM cells were seeded at a density of 20 × 10^6^ per Ø 60 mm Petri dishes in full culture medium. The digital images of fixed randomly selected view fields were acquired using a phase-contrast microscopy (Nikon Eclipse TiU, Nikon Corporation, Tokyo, Japan) equipped with CCD-camera on day 4 and day 14. The number of cells was calculated using the Sigma Scan Pro 5 program (SPSS, USA). Proliferative activity was assessed as population doubling (PD). PD was estimated as follows: PD = [log (N/N0)]/log2, where N0 and N were initial and final number of cells, respectively [41].

### 4.6. Osteogenic Differentiation

To assess the level of spontaneous osteocommitment and response to osteoinduction, the first passage MMSCs were seeded at a density of 2 × 10^3^/cm^2^ in 35 mm Petri dishes and cultured in full culture medium for 7 days. Then, a culture medium in a half of culture dishes was replaced with Mesenchymal Stem Cell Osteogenesis Kit (Millipore, Temecula, CA, USA) to induce osteodifferentiation. On day 7, a part of dishes in both control and induced groups were used for histochemical detection of activity of early marker of osteodifferentiation—alkaline phosphatase with Alkaline Phosphatase Kit (Sigma-Aldrich Corp., St. Louis, MO, USA). Briefly, cells were fixed with 4% paraformaldehyde for 30 min at room temperature, washed thoroughly with PBS and stained with solutions from Alkaline Phosphatase Kit (Sigma-Aldrich Corp., St. Louis, MO, USA) for 30 min according to the manufacturer’s instructions.

**Figure 6:**
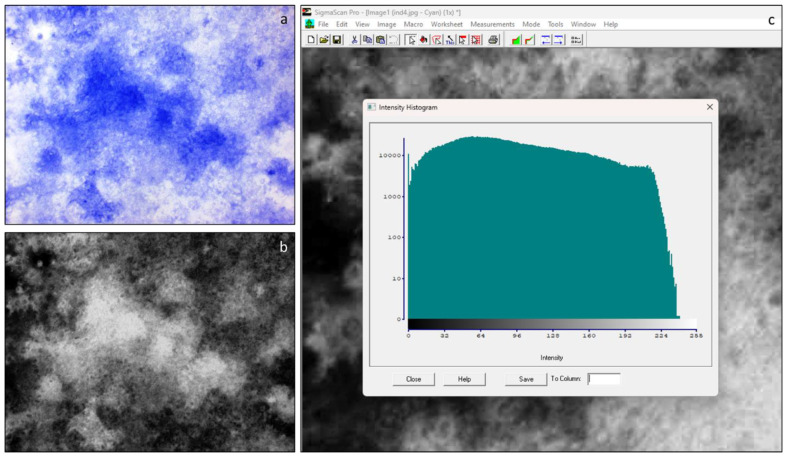
Semiquantitative evaluation of alkaline phosphatase activity. ALP activity was characterized by the presence of the colored product of histochemical reaction (**a**). The different tones of blue were converted into greyscale with Sigma Scan Pro 5 software (**b**). Representative histogram of color intensity distribution. The intensity of staining was divided into 256 channels and measured in each channel (**c**).

The activity of enzyme was characterized by the presence of the colored product, and the intensity of staining was examined with bright field microscopy (Nikon Eclipse TiU, Nikon Corporation, Tokyo, Japan). Subsequent processing of digital images was done using the Sigma Scan Pro 5 software. The colored product had different tones of blue, and the intensity of the staining reflected the activity of the enzyme. The intensity of blue staining was divided into 256 channels and measured in each channel (Figure 6). In our samples, stained cells fell into channels 1 through 80. On the basis of these data, histograms of color intensity distribution were plotted.

On day 21, the mineralization of extracellular matrix (ECM) as a sign of late osteodifferentiation was assessed using Alizarin Red staining (Sigma-Aldrich Corp., St. Louis, MO, USA). The cells were fixed with 4% paraformaldehyde for 30 min at room temperature, and incubated with alizarin red (2% aqueous solution, pH 4.1–4.3, adjusted with ammonium hydroxide) for 30 min. Excess of stain was removed by washing four times with water. To determine the area occupied by the mineralized ECM, the entire culture surface was photographed using a Stemi 2000-C stereoscopic microscope (Carl Zeiss, Oberkochen, Germany), after which the image was analyzed using Sigma Scan Pro 5 software (SPSS, Point Eichmond, CA, USA).

### 4.7. Paracrine Activity

The soluble mediators were detected in conditioned medium of MMSCs using multiplex analysis with Luminex xMAP technology. We used Milliplex xMAP system and cytokine assay kit for IL-1a, IL-4, IL-1b, IL-2, IL-6, IL-13, IL-10, IL-12p70, IFNg, IL-5, IL-17A, IL-18, IP-10, TNFa, as well as regulators of bone tissue metabolism: ACTH, OPG, Insulin, Leptin, PTH, DKK1, SOST, FGF23 (Merck Millipore, Burlington, MA, USA) according to manufacturer’s protocol.

### 4.8. Quantitative Real-Time PCR

To determine the level of gene expression, total RNA was isolated using QIAzol lysis reagent (Qiagen Sciences LLC, Germantown, MD 20874, USA), followed by a reverse transcription reaction using QuantiTect Reverse Transcription Kit (Qiagen Sciences LLC, MD 20874, USA) according to the manufacturer’s instructions. The obtained cDNA was used for quantitative PCR using a commercial RT^2^—Real Time SYBR Green/ROX PCR master mix (Qiagen Sciences LLC, MD 20874, USA) and a set of lyophilized primers (Mmp9, Spp1, Cxcl12, Ppar γ, Runx2, RANKL, OPG, Ibsp, BMP10, Cxcr4, Sost, Alpl) QuantiTect Primer Assays (Qiagen Sciences LLC, MD 20874, USA). Normalization was performed according to the expression level of the reference gene Rplp1 (Qiagen Sciences LLC, MD 20874, USA). The expression level was assessed using the 2^−∆∆Ct^ method according to the manufacturer recommendation (Qiagen Sciences LLC, MD 20874, USA).

### 4.9. Statistical Analysis

For the ex vivo experiments three independent stromal cell lines isolated from each single BM were used. Three technical repetitions were run in each experimental set. Ten randomly selected view fields were examined in morphometric assays.

Statistical analysis was performed using Microsoft Excel 2010 and Statistica 7.0, with analysis of variance method. Data are presented as mean +/− SEM. The differences were considered significant at *p* < 0.05.

## Figures and Tables

**Table 1:** Cellularity of rat hindlimb bone marrows.

Experimental Group	Femur	Tibia
VC	326 ± 65	193 ± 25
HU	149 ± 23 *	112 ± 3 *^#^
HU + RL	173 ± 17 *	150 ± 12 *

The data are presented as mean + SEM of total number of nucleated cells (TNCs)/10^6^ per bone marrow sample (*n* = 3). * *p* < 0.05, the significant difference from VC. ^#^ I < 0.05, the significant difference from HU + RL. VC—Vivarium Control; HU—Hindlimb Unloading; HU + RL—Reloading after HU.

**Table 2:** Population doublings (PD) in MMSC cultures from rat hindlimb bone marrow.

Exp. Group	Femur	Tibia
VC	3.9 ± 0.4	3.6 ± 0.4
HU	1.4 ± 0.5 *^#^	1.2 ±0.3 *^#^
HU + RL	3.6 ± 0.2	3.6 ± 0.2

The data are presented as mean + SEM (*n* = 3). * *p* < 0.05, the significant difference from VC. ^#^ *p* < 0.05, the significant difference from HU + RL. VC—Vivarium Control; HU—Hindlimb Unloading; HU + RL—Reloading after HU.

**Table 3:** Paracrine activity of MMSCs from rat hindlimb bone marrows.

BM Source	Femur			Tibia		
	Experimental Groups					
Level, pg/mL	VC	HU	HU + RL	VC	HU	HU + RL
IL-5	15.3 ± 1.4	17.5 ± 3.9	14.0 ± 5.8	15.3 ± 1.2	20.0 ± 5.5	14.0 ± 6.1
IL-6	155.2 ± 46.7	2971.0 ± 186.4 *^#^	79.2 ± 24.4 *	141.6 ± 35.5	1314.5 ± 142.8 *^#^	353.7 ± 59.7 *
IL-10	47.8 ± 3.6	124.1 ± 11.1 *^#^	14.0 ± 2.7 *	69.8 ± 15.6	99.6 ± 7.5 *^#^	41.8 ± 8.3
IL-13	15.4 ± 1.9	17.3 ± 1.4	13.6 ± 5.1	14.8 ± 1.4	13.6 ± 1.1	0
IP-10	213.1 ± 28.6	301.8 ± 24.7 *	360.2 ± 36.6 *	238.0 ± 25.7	360.3 ± 30.2 *	360.0 ± 20.1 *
TNF-a	0	5.6 ± 1.1 *^#^	0	0	3.5 ± 1.2 *^#^	0
OPG	113.0 ± 28.3	57.3 ± 1.6 *^#^	188.1 ± 1.2 *	88.8 ± 9.4	59.1 ± 7.0 *^#^	113.7 ± 12.1 *
SOST	172.5 ± 13.0	50.4 ± 8.6 *	67.7 ± 6.83 *	383.7 ± 74.8	94.6 ± 23.1 *^#^	322.7 ± 55.8

The data are presented as median of 3 independent experiments in 2 technical replications. *—significant difference from VC (*p* ≤ 0.05). ^#^ *p* < 0.05, the significant difference from HU + RL. VC—Vivarium Control; HU—Hindlimb Unloading; HU + RL Reloading after HU.

## Data Availability

The data presented in this study are available on request from the corresponding author. The data are not publicly available due to Institution policy.
